# *Eimeria zuernii* (Eimeriidae: Coccidia): mitochondrial genome and genetic diversity in the Chinese yak

**DOI:** 10.1186/s13071-023-05925-8

**Published:** 2023-09-03

**Authors:** Xuan Zhou, Zhao Wang, Pengchen Zhu, Xiaobin Gu, Ran He, Jing Xu, Bo Jing, Lidan Wang, Shun Chen, Yue Xie

**Affiliations:** 1 Department of Parasitology, College of Veterinary Medicine, Sichuan Agricultural University, Sichuan, 611130 China; 2 Institute of Preventive Veterinary Medicine, Sichuan Agricultural University, Sichuan, 611130 China

**Keywords:** Yak coccidiosis, *Eimeria zuernii*, MtDNA, Genetic relationships, Population structure

## Abstract

**Background:**

Coccidiosis caused by *Eimeria zuernii* (Eimeriidae: Coccidia) represents a significant economic threat to the bovine industry. Understanding the evolutionary and genetic biology of *E. zuernii* can assist in new interaction developments for the prevention and control of this protozoosis.

**Methods:**

We defined the evolutionary and genetic characteristics of *E. zuernii* by sequencing the complete mitogenome and analyzing the genetic diversity and population structure of 51 isolates collected from eight yak breeding parks in China.

**Results:**

The 6176-bp mitogenome of *E. zuernii* was linear and encoded typical mitochondrial contents of apicomplexan parasites, including three protein-coding genes [PCGs; cytochrome *c* oxidase subunits I and III (*cox*1 and *cox*3), and cytochrome b (*cyt*b)], seven fragmented small subunit (SSU) and 12 fragmented large subunit (LSU) rRNAs. Genome-wide comparative and evolutionary analyses showed *cyt*b and *cox*3 to be the most and least conserved *Eimeria* PCGs, respectively, and placed *E. zuernii* more closely related to *Eimeria mephitidis* than other *Eimeria* species. Furthermore, *cox*1-based genetic structure defined 24 haplotypes of *E. zuernii* with high haplotype diversities and low nucleotide diversities across eight geographic populations, supporting a low genetic structure and rapid evolutionary rate as well as a previous expansion event among *E. zuernii* populations.

**Conclusions:**

To our knowledge, this is the first study presenting the phylogeny, genetic diversity, and population structure of the yak *E. zuernii*, and such information, together with its mitogenomic data, should contribute to a better understanding of the genetic and evolutionary biological studies of apicomplexan parasites in bovines.

**Graphical Abstract:**

**Figure:**
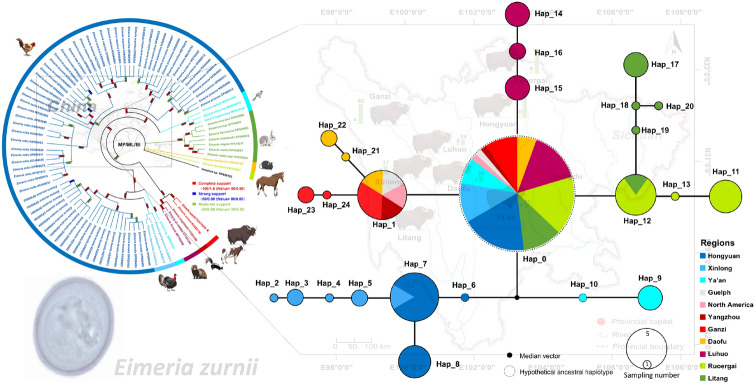

**Supplementary Information:**

The online version contains supplementary material available at 10.1186/s13071-023-05925-8.

## Background

Bovine coccidiosis, caused by *Eimeria* spp., is a widespread and highly pathogenic protozoosis with a significant economic impact on the cattle industry [1]. Similar to that in cattle, coccidiosis is also commonly found in yaks, and there are 17 species of the genus *Eimeria* responsible for this disease, including *E. zurnii*, *E. bovis*, *E. auburnensis*, *E. canadensis*, *E. ellips*, *E. alabamensis*, *E. bareillyi*, *E. brasiliensis*, *E. bukidnonensis*, *E. cylindrica*, *E. kwangsiensis*, *E. mandela*, *E. pellita*, *E. subspherica*, *E. wyomingensis*, *E. illinoisensis*, and *E. stiedai*-like [1, 2]. *Eimeria zuernii* is by far the most pathogenic coccidian species and poses an additional mortality threat to yak calves < 1 year old [2–4]. Infection with *E. zuernii* can cause malabsorption of nutrients, watery or bloody diarrhea, and even death in heavily infected animals [5].

China has the largest population of the yak in the world, with 16 million yaks accounting for > 95% of the global population [6]. Recent epidemiological evidence, however, showed that *E. zuernii* is commonly prevalent in yak farms and is becoming the leading cause of weight reduction in young individuals across the Qinghai-Tibetan Plateau of China [5, 7–10]. Such situations highlight the significance and necessity of the diagnosis and control of *E. zuernii*. Current diagnosis and identification of this protozoan parasite typically rely on morphological characteristics. Unfortunately, *E. zuernii* has similar oocyst morphotypes and overlapping biological features with other coccidian species. In such a context, obtaining an efficient approach to identify *E. zuernii* infections becomes urgent for clinical diagnosis, epidemiological investigation, and control, and achieving this goal is foreseeable only through the utilization of molecular approaches. Although the internal transcribed spacer 1 (ITS-1) region and small subunit ribosomal DNA (SSU) of the nuclear ribosomal DNA (rDNA)-based PCR amplifications were applied to differentiate between bovine coccidian species, including *E. zuernii* [11–13], cumulative studies demonstrated that the mitogenomic DNA (mtDNA) seems to be more powerful in delimiting individual *Eimeria* species because of its rapid evolution rate, absence of recombination, and matrilineal inheritance [14]. Furthermore, Awadi et al. showed that the *Eimeria* mitogenomes have the ability to illustrate their evolution and host adaptations [15]. Therefore, it is reasonable that the mitochondrial (mt) datasets are widely used for species-specific identification in bovine coccidiosis [16–20]. Unfortunately, until now, no complete information on the mitogenome of *E. zuernii* has been characterized. Additionally, a fundamental understanding of the genetic structure and diversity of *E. zuernii* would also assist in the development of a long-lasting control strategy. Several in-depth studies have proven their significance and effectiveness in controlling protozoan *Plasmodium* spp. and *Toxoplasma* spp. in humans as well as *Eimeria* spp. in chickens by defining their genetic diversity and population architecture [21–23]. Of course, numerous factors, including the effective population size, distance between populations, host dispersal ability, evolutionary history, host population structures, and the complexity of the life cycle, may affect the genetic makeup of *E. zuernii* populations [24–26]. However, little is known about the genetic diversity and population structure of *E. zuernii* so far. Herein, we decoded the complete mitogenome of *E. zuernii* isolated from Chinese yaks and further determined its population genetic diversity by sequencing the *cox*1 gene of 51 *E. zuernii* isolates that were collected from different geographical yak breeding parks in Sichuan, China. These results would add novel insights to phylogenetic, population genetic, and molecular epidemiological studies on this yak parasite.

## Methods

### Sample collection

**Fig. 1:**
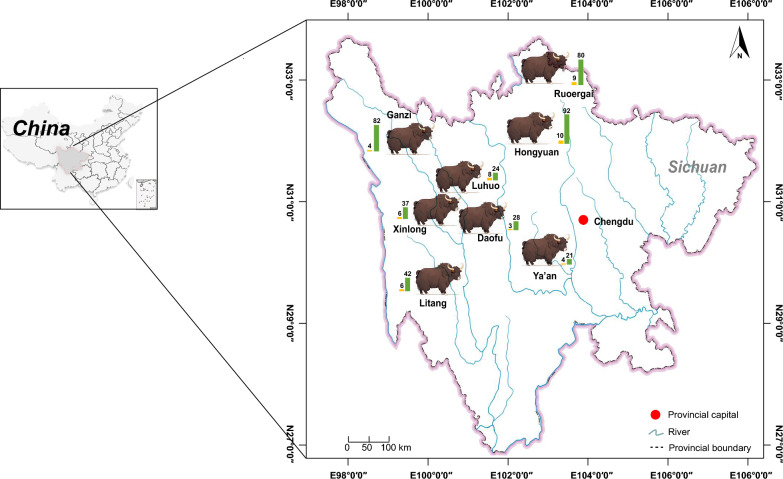
Map of sampling localities for yak *Eimeria* isolates included in the present study. The number of isolates infected with *E. zuernii* (yellow bars) and other *Eimeria* species (green bars) from each locality are shown, respectively

In November 2022, as part of ongoing surveys of coccidiosis in yaks, 456 *Eimeria* oocyst-positive fecal samples were obtained from eight Yak Modern Industrial Parks in Sichuan, China: 43 in Xinlong, 102 in Hongyuan, 25 in Ya’an, 89 in Ruoergai, 32 in Luhuo, 48 in Litang, 31 in Daofu, and 86 in Ganzi (Fig. 1). For species identification, about 500-g feces from each sampling were mashed and suspended with running water, followed by filtration through a series of sieves (300, 150, and 80 mm; Endecotts, London, UK) and culture in a 5% (w/v) potassium dichromate solution at 28 °C for 72 h under forced aeration. Sporulated oocysts were speciated according to morphometric keys [27]. Of 456 *Eimeria* isolates, 51 were preliminarily identified as pure infections with *E. zuernii* (Additional file 1: Table S1), and their oocysts were further subjected to enrichment with saturated sodium nitrate flotation and molecular identification by PCR amplification using the species-specific primers (forward: 5′-CCCACTACATCCAACCTCCTG-3′; reverse: 5′-GCGTTCGGAAATCTGATGGT-3′) [9] and sequence comparisons with the targeted ITS-1 regions. All isolates were determined to share > 99.2% identities with the query sequence of *E. zuernii* in GenBank (Additional file 2: Fig. S1; accession nos. OR351547–OR351597 vs. AB769665).

### Sequencing and assembly

After species identification and oocyst quantitation using a McMaster chamber among these *E. zuernii* isolates, one sample from Ganzi was selected for mitogenome sequencing because of its high number of oocysts per gram (OPG = 3.8 × 10^6^). About 2.5 million *E. zuernii* oocysts were harvested and purified by using a combination of saturated sodium nitrate flotation and discontinuous sucrose gradient centrifugation. Following vortex breakdown, the genomic DNA was extracted from oocysts using the Genomic DNA Kit (Tiangen, Beijing, China). Based on the quality and quantity assessment, ~ 2.5 µg genomic DNA was fragmented to construct a 250-bp paired-end (PE) library, followed by sequencing on an Illumina HiSeq X-TEN platform (BerryGenomics, Beijing, China). The clean reads (~ 1.8 Gb) were assembled with MITObim v1.9.1 [28]. The genome was validated by PCR amplifications using four overlapping fragments (Table 1) and then annotated using MITOS2 (http://mitos2.bioinf.uni-leipzig.de/index.py) and a whole genome-guided gene alignment against *Eimeria mephitidis* (accession no. KT203398). Based on the *E. zuernii* mitogenome sequenced here, a pair of primers, *cox*1-F (5′-TTGGTTGGACTCTATACCCTC-3′) and *cox*1-R (5′-AGATAGTACAAAATGAAAATGAGC-3′), were designed to amplify the *cox*1 gene (776 bp) from the remaining 50 *E. zuernii* isolates. PCR reactions (50 μl) containing 2 μl of template genomic DNA, 2 μl of each primer (10 pmol each), 25 μl of 2 × Taq MasterMix (TIANGEN, Beijing, China), and 19 μl of ddH_2_O were performed in a S1000 Thermal Cycler (Bio-Rad, USA) using the following conditions: initial denaturation at 94 °C for 5 min; 35 cycles of 94 °C for 1 min, 53 °C for 30 s, 72 °C for 30 s; followed by a final extension at 72 °C for 8 min. Target bands were isolated by 1.0% agarose-TAE gel electrophoresis and purified using spin columns (Wizard PCR Prep, Promega, USA). The purified amplicons were cloned into the vector pMD19-T (TakaRa, Dalian, China), and each clone was sequenced three times on an automatic DNA sequencer by Shenggong Biological Technology Company (Shanghai, China). The complete mitogenome sequence of *E. zuernii* and sequences of *cox*1 (776 bp) in 50 *E. zuernii* isolates were deposited in GenBank under accession numbers OQ476205 and OR039219–OR039268.

**Table 1:** Primer pairs for PCR amplification and their positions in the *Eimeria zuernii* mitogenome

Primer name	Location based on *E. zuernii* mitogenome	Primer sequence (5′ to 3′)
Ez-1	1–20	Forward: GATAAAGTCGCAGCAGTAGC
	1648–1672	Reverse: AGGTTGGAGAGAGTGACATTAGAGA
Ez-2	934–950	Forward: TATTCCAAGTAAAGCTG
	2892–2912	Reverse: AACCCAGCTCACGTATCACAT
Ez-3	2835–2850	Forward: GCTCATCACACCCTTG
	5090–5112	Reverse: CTTAACCCAGCTCACGTATCACA
Ez-4	5039–5060	Forward: AAGACAGAATAGTATTTTTTAA
	511–532	Reverse: AGTAGCACCCCAGAAGCTCATT

### Sequences analysis

Nucleotide composition and codon usage of the *E. zuernii* mitogenome were measured with MEGA X (www.megasoftware.net). The base skewnesses of different mitogenomic regions of *E. zuernii* were determined using the formulas AT skew = (A − T)/(A + T) and GC skew = (G − C)/(G + C) [29]. The multi-alignments of nucleotide and amino acid sequences of protein-coding genes (PCGs) obtained from the sequenced mtDNA and those of other available *Eimeria* parasites (Additional file 3: Table S2) were achieved using MEGA X. Based on the alignments, the similarities and divergences of PCGs were determined using DNAstar v5.02 (DNAStar Inc., Madison, WI), genetic distances were calculated based on Kimura-2-parameter (K2P) [30] with MEGA X, and the ratio of the nonsynonymous substitution (Ka) and synonymous substitution (Ks) of each PCG was calculated with KaKs_Calculator [31].

### Phylogenetic and population structure analyses

The phylogenetic position of *E. zuernii* and relationships between different host-originated eimerian parasites in the genus *Eimeria* were inferred from the complete mitogenomes of *E. zuernii* and other congeneric species (Additional file 3: Table S2), using *Isospora* sp. (accession no. KP658103) as the outgroup. After nucleotide alignments using MAFFT v7.450 [32] and filtration of the ambiguous regions using Gblocks v0.91b (http://molevol.cmima.csic.es/castresana/Gblocks.html), a 6552-bp multi-sequence dataset was built to reconstruct the phylogenetic tree with three algorithms, including maximum parsimony (MP), maximum likelihood (ML), and Bayesian inference (BI). For the MP analysis, the tree was constructed by PAUP v4.0b10 [33] using equally weighted parsimony and heuristic searches and tree-bisection-reconnection (TBR) branch-swapping, and the optimal topology was obtained using the Kishino-Hasegawa method with 1000 replicates. The ML analysis was implemented using PHYML v3.1 [34] under the optimal evolutionary model “TIM + F + I + G4” that was selected with the “Auto” option on the W-IQ-TREE web server (http://iqtree.cibiv.univie.ac.at) using an ML + rapid bootstrap algorithm with 1000 replicates. The BI analysis was achieved by MrBayes v3.2.7 [35] using the optimal evolutionary “CAT + GTR + G” model chosen by ModelFinder [36] and four independent Markov chain Monte Carlo (MCMC) chains running for 1,000,000 generations; after sampling a tree every 0.1% generation, a consensus tree was obtained and visualized in Treeview X (https://www.linuxlinks.com/treeviewx/). In parallel, 50 *E. zuernii* isolates sampled from eight different geographical yak breeding parks were subjected to an assessment of population diversity using the *cox*1-based mt dataset. Diversity indices for these *E. zuernii* isolates, including the number of haplotypes, haplotype diversity, and nucleotide diversity, were calculated in DnaSP v6.12 (http://www.ub.edu/dnasp/). A phylogeny of haplotypes was estimated by MP tree using MEGA X, with the significance of each node estimated using 10,000 bootstrap replicates of the dataset. The neutrality indices, including Fu’s *Fs*, Tajima’s *D*, and the pairwise fixation index (Fst), were used to estimate the size variation among populations using Arlequin v3.5.2.2 (http://cmpg.unibe.ch/software/arlequin35/). The median-joining networks among sequences of populations from eight geographic ranges were drawn using Population Analysis with Reticulate Trees (PopART; http://popart.otago.ac.nz) to reflect whether the genetic differentiation between populations was associated with geographical isolations.

## Results and discussion

### *Eimeria zuernii* mitogenome feature

**Fig. 2:**
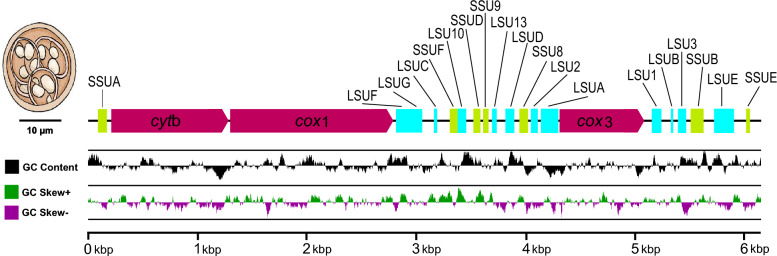
Linear organization of *E. zuernii* mtDNA. The order and transcriptional direction of three coding regions for *cyt*b, *cox*1, and *cox*3 are indicated by purple arrows and fragments of seven SSU (green) and 12 LSU (light blue) rDNAs are detected between the protein-coding regions and identical to those of other congeneric species. The nomenclature of these ribosomal fragments follows the convention of Feagin et al. (2012) [40]. Positive and negative GC-skew and GC content are indicated in dark green, purple, and black, respectively, across the whole genome

The *E. zuernii* linear mitogenome was 6176 bp in size and encoded three PCGs, *cyt*b, *cox*1, and *cox*3, as well as seven interspersed small subunit (SSU) and twelve interspersed large subunit (LSU) rDNA fragments (Fig. 2). No transfer RNAs (tRNAs) were found in the *E. zuernii* mitogenome, similar to other *Eimeria* spp. [17, 37–43]. Likewise, the *E. zuernii* mitogenome was biased towards AT (64.52–67.37%) with T as the most favored base and G the least favored. Across the mitogenome, there were three overlapping regions located between *cyt*b and *cox*1 genes (4 bp), between LSUF and LSUG rRNA genes (7 bp), and between LSUA rRNA and *cox*3 genes (6 bp), respectively. Moreover, 18 intergenic spacers were also observed, ranging in size from 9 to 221 bp, in the *E. zuernii* mitogenome (Additional file 4: Table S3).

**Fig. 3:**
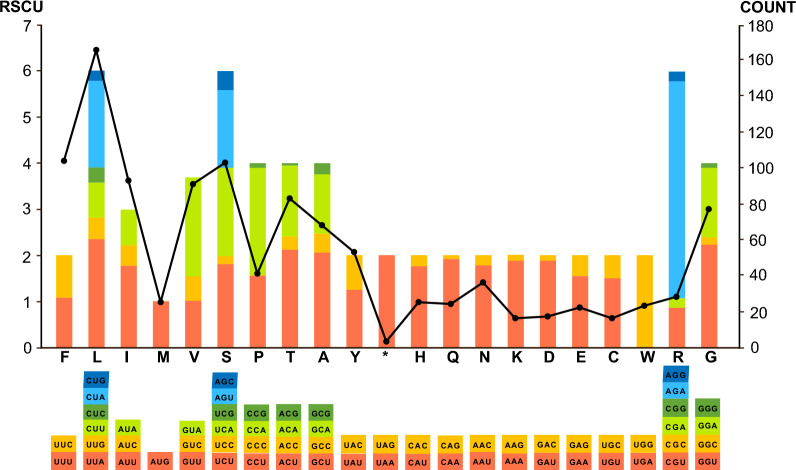
Codon usage in PCGs of *E. zuernii* mtDNA. The numbers on the left and right *Y*-axis scales referred to the RSCU value and the total number of codons, respectively. Codon families were plotted under the *X*-axis scale and represented by different colors. The codon counts were depicted by the black line graph

Within three PCGs, *cox*1 was presented between *cyt*b and LSUF, *cox*3 between LSUA and LSU1, and *cyt*b between SSUA and *cox*1, respectively, with obvious AT skewness (AT-skew =  − 0.17; Additional file 5: Table S4). Such AT skewness was also reflected in their codon usage patterns and relative synonymous codon usage (RSCU) of PCGs. As shown in Fig. 3, the most frequently used codon of the PCGs was AGA (RSCU = 4.71), followed by UUA (RSCU = 2.35), CCA (RSCU = 2.34), and GGU (RSCU = 2.15), corresponding to the most frequently used amino acid Leu (*n* = 166), followed by Phe (*n* = 104) and Ser (*n* = 103), similar to other *Eimeria* species. Furthermore, among the start codon choices, except for the *cox*3 gene, which was deduced to use a non-standard start codon TTG, the *cox*1 and *cyt*b genes used ATA and ATG as the start codons, respectively. TAA was used as the stop codon to terminate the *cyt*b, *cox*1, and *cox*3 genes. For SSU rDNAs, seven gene fragments included SSUA, SSUB, SSUD, SSUE, SSUF, SSU8, and SSU9 and ranged in size from 37 (SSUF) to 116 bp (SSUB). Within LSU rRNAs, 12 gene fragments included LSUA, LUSB, LUSC, LSUD, LSUE, LSUF, LUSG, LSU1, LSU2, LSU3, LSU10, and LSU13 and ranged in size from 16 (LUSC) to 188 bp (LSUE). It appeared that these fragmented rRNAs were more conserved than PCGs, possibly related to their functional constraints in the former [39].

### Comparative mitogenomics among *Eimeria*

To understand the evolutionary divergence of *Eimeria* spp., the interspecific variations between and among *E. zuernii* and congeneric species were determined using the nucleotide and amino acid sequences of single and concatenated mt PCGs. The interspecific variations for each PCG ranged from 11.4% to 29.4% at the nucleotide level and 6.4% to 23.5% at the amino acid level. Furthermore, on the basis of the concatenated PCGs, the interspecific variations ranged from 14.0% to 23.2% for nucleotide sequences and 9.0% to 16.3% for amino acid sequences (Additional file 6: Table S5). It was obvious that the highest sequence variation was found in the *cox*3 gene while the lowest sequence divergence was found in the *cyt*b gene, suggesting *cyt*b was the most conserved PCG among *Eimeria* mitogenomes, which is consistent with previous findings in species of *Babesia*, *Theileria*, and *Plasmodium* [44, 45]. Moreover, it also seemed noteworthy that regardless of nucleotide or amino acid levels, *E. zuernii* always shared the lowest sequence variation with *Eimeria mephitidis*, to some extent, suggesting their closer genetic similarity than other *Eimeria* species.

### Evolutionary and phylogenetic analyses

**Fig. 4:**
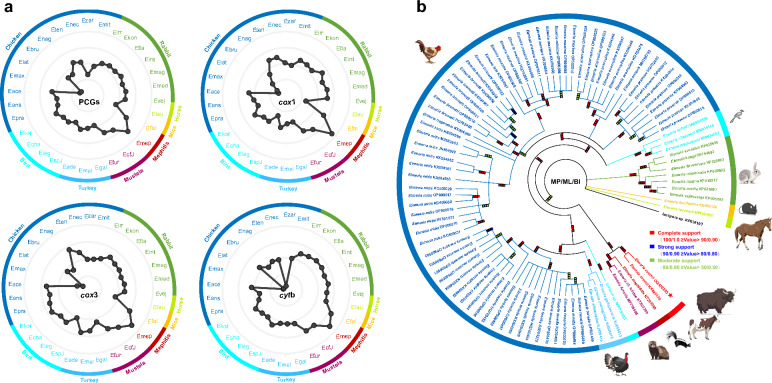
Evolutionary and phylogenetic relationships between *E. zuernii* and other congeneric species. **a** Patterns of K2P distance between *Eimeria zuernii* and other 30 *Eimeria* species determined on the basis of single and concatenated mt PCGs. Gray lines indicate the same K2P distance from the center. Black dots show the relative K2P distances between *E. zuernii* and other eimerian parasites. Dots closer to the edge of the patterns indicate a smaller K2P distance between *E. zuernii* and the corresponding species. Species abbreviations: *E. anseris*, Eans; *E. chapmani*, Echa; *E. kofoidi*, Ekof; *E. legionensis*, Eleg; *Eimeria* sp._JRBarta-2021b, Espj; *E. acervuline*, Eace; *E. brunetti*, Ebru; *E. lata*, Elat; *E. maxima*, Emax; *E. mitis*, Emit; *E. nagambie*, Enag; *E. necatrix*, Enec; *E. praecox*, Epra; *E. tenella*, Eten; *E. zaria*, Ezar; *Eimeria* cf._ictidea_JRB-2016, Ecfi; *E. falciformis*, Efal; *E. furonis*, Efur; *E. leuckarti*, Eleu; *E. mephitidis*, Emep; *E. flavescens*, Efla; *E. intestinalis*, Eint; *E. irresidua*, Eirr; *E. kongi*, Ekon; *E. magna*, Emag; *E. media*, Emed; *E. vejdovskyi*, Evej; *E. adenoeides*, Eade; *E. gallopavonis*, Egal; *E. meleagridis*, Emel. **b** Phylogenetic tree inferred by MP, ML, and BI algorithms using complete mitogenome datasets of *E. zuernii* and other congeneric species. *Eimeria* species, together with their branches, are marked with different colors according to host origins as follows: bird in light blue, chicken in dark blue, horse in light green, mephitis in dark red, mouse in yellow, mustela in purple, rabbit in green, turkey in blue, and yak/cattle in red. *Eimeria zuernii* sequenced in this study is indicated in bold font along with a star. The color boxes at each node showed bootstrap values for MP/ML/BI

To measure the interspecific genetic similarity between *E. zuernii* and other *Eimeria* spp., single or concatenated mt PCGs were also used to calculate genetic distances among *Eimeria* based on the K2P model [30]. As shown in Fig. 4a, based on either single or concatenated mt PCG datasets, it was clear that the minimum K2P values were always present between *E. zuernii* and *E. mephitidis* (0.114 and 0.139) in contrast with the maximum values present between *E. zuernii* and *Eimeria maxima* (0.210 and 0.173), supporting that *E. zuernii* was closely related to *E. mephitidis* but diverged from *E. maxima*. Furthermore, among genetic distance structures, the *cox*3-based K2P values were all obviously larger than those of the *cyt*b, *cox*1, and concatenated PCGs, in agreement with the aforementioned result in which *cox*3 was determined to be the most divergent gene among *Eimeria* PCGs. In parallel with genetic distance analysis, the selective pressure placed on mt PCGs of *Eimeria* during evolution was determined by qualification of the Ka, Ks, and Ka/Ks ratios (Additional file 7: Table S6). Our results showed that all Ka/Ks values were < 1, suggesting that three PCGs were subjected to negative or purifying selection, to a certain extent, in agreement on the conservation and validity of the genus *Eimeria* in the course of evolution.

The phylogenetic relationships among eimerian parasites were inferred based on the complete mitogenomic dataset (containing 4315 conserved and 2237 variable sites) of *Eimeria* spp. available in GenBank (Additional file 3: Table S2). As shown in Fig. 4b, three phylogenetic trees (MP/ML/BI) consistently supported a paraphyletic relationship among species of *Eimeria*, and this relationship can be marked according to their host origins, except for bird-infecting species, consistent with previously conducted phylogenetic studies [46–49]. Notably, *E. zuernii* was placed close to *E. mephitidis* and together grouped with *Eimeria* cf. *ictidea* and *Eimeria furonis* as a clade (Clade I), with high statistical support (all statistical values ≥ 99 or 0.99). Furthermore, this clade was more closely related to *Eimeria* species of birds (Clade II) than to *Eimeria* species of rabbits (Clade III), in accordance with recent mtDNA-based phylogenetic conclusions [18, 19, 41, 50]. It appeared that these three clades occupied the main part of the phylogenetic tree, although their topological relationships with *Eimeria* species of mice and horses remained to be determined because of the poor bootstrap supports observed here (Fig. 4b). The previous mt *cox*1-based phylogenetic analysis showed that the *E. zuernii*-contained clade was more closely related to mouse *Eimeria* spp. than to bird *Eimeria* spp.; however, the nuclear 18S-based phylogeny indicated a closer relationship between *E. zuernii*-contained clade and rabbit *Eimeria* spp. than between *E. zuernii*-contained clade and bird *Eimeria* spp. [51]. This discordance in the mt and nuclear phylogenies is surprising and might be due to a greater rate of nucleotide change in the mitogenomes of eimerian parasites than that seen in nuclear-encoded sequences, as reported in helminths [52]. Therefore, future studies using mitogenomic data from more widespread species or isolates of *Eimeria* from herbivorous and omnivorous animals worldwide are required to determine the evolutionary relationships within the entire genus *Eimeria*.

### Population structure

The number of haplotypes, haplotype diversity value Hd, and nucleotide diversity π of each *E. zuernii* population were calculated and are shown in Table 2. The Xinlong population had the highest Hd value (0.867) and π (0.00326), while the Ya’an population had the lowest Hd value (0.500) and π (0.00064). Ganzi and Litang populations had the same Hd value of 0.800, whereas the Litang population had a lower π value than the Ganzi population (0.00163 vs. 0.00232). Likewise, Daofu and Ruoergai populations had the same Hd value of 0.667, whereas the Daofu population had a lower *π* value than of the Ruoergai population (0.00086 vs. 0.00143). For the Luhuo population, the respective Hd value was 0.750 and *π* was 0.00138, and for the Hongyuan population, the Hd value was 0.644 and *π* was 0.00097. Combined, these results showed that these eight *E. zuernii* populations had high haplotype diversities and low nucleotide diversities. Similar phenomena were seen in other protozoan parasites with large standing population sizes and extremely high fecundities, including *Trichomonas vaginalis*, *Giardia duodenalis*, and *Theileria annulate* [53–55], which might reflect a high matrilineal effective population size for *E. zuernii* and also signify the occurrence of expansion of a low effective population size of *E. zuernii* after a period because of the rapid population growth enhancing the retention of new regions.

**Table 2:** Summary of the genetic diversity of eight geographic populations of *E. zuernii* on the basis of the *cox*1 dataset

Population	No. of individuals	No. of haplotypes	Haplotype diversity (Hd)	Nucleotide diversity (*π*)	Fu’s *Fs*	Tajima’s *D*
Hongyuan	10	3	0.644	0.00097	− 0.046	0.222
Xinlong	6	4	0.867	0.00326	− 0.071	0.878
Ya’an	4	2	0.500	0.00064	0.172	− 0.612
Ganzi	5	3	0.800	0.00232	0.469	1.573
Daofu	3	2	0.667	0.00086	0.172	− 0.612
Luhuo	8	3	0.750	0.00138	0.330	1.449
Ruoergai	9	3	0.667	0.00143	0.551	1.754
Litang	6	4	0.800	0.00163	− 1.350	− 0.185

In addition, the negative values of Fu’s *Fs* were observed in Hongyuan (*F*s =  − 0.046), Xinlong (*F*s =  − 0.071), and Litang (*F*s =  − 1.350) populations, in contrast with the positive values of Fu’s *Fs* found in Daofu (*F*s = 0.172), Ya’an (*F*s = 0.172), Luhuo (*F*s = 0.330), Ganzi (*F*s = 0.469), and Ruoergai (*F*s = 0.551) populations (Table 2). Likewise, the negative value of Tajima’s *D* was also observed in the Litang (*D* =  − 0.185) population, in contrast with the positive values of Tajima’s *D* found in Luhuo (*D* = 1.449), Ganzi (*D* = 1.573), and Ruoergai (*D* = 1.754) populations (Table 2). Although the differences among populations in both neutrality tests were not significant, the negative values of the two neutrality indices in the Litang population indicated that the population had undergone expansion, while positive values in the Luhuo, Ganzi, and Ruoergai populations suggested these populations experienced a bottleneck [56–58]. Furthermore, significantly high Fst values were also observed between populations (Additional file 8: Table S7). The highest Fst value was seen between Hongyuan and Daofu populations (Fst = 0.92157, *P* < 0.05), followed by Fst values between Hongyuan and Ya’an populations (Fst = 0.91220, *P* < 0.01), between Hongyuan and Litang populations (Fst = 0.89787, *P* < 0.05), between Luhuo and Daofu populations (Fst = 0.88665, *P* < 0.05), between Ruoergai and Daofu populations (Fst = 0.88406, *P* < 0.05), between Ya’an and Luhuo populations (Fst = 0.88360, *P* < 0.05), and between Ya’an and Ruoergai populations (Fst = 0.88066, *P* < 0.01). Such high Fst values indicated a possible geographical isolation of *E. zuernii*. Indeed, when taking the yak’s features, including the free-ranging, high-altitude grazing, and self-reproduction model, as well as the captive history of individuals sampled here into account, it is possible that the lack of translocations and/or introductions of these host populations between parks contributes to this population isolation of *E. zuernii*. Interestingly, such high Fst values were also observed in other protozoan parasites, such as *G. duodenalis* and *Cryptosporidium* spp., which were sampled from self-breeding dairy farms [55, 57]. To some extent, this high Fst value supports the phenomenon that the low gene flow and high genetic diversity of the bovine protozoa could be accelerated in an independent self-service farming mode. Of course, the phenomenon remains further validated when more additional protozoan population genetic data become available, especially those from hosts bred in an independent self-service farming model.

**Fig. 5:**
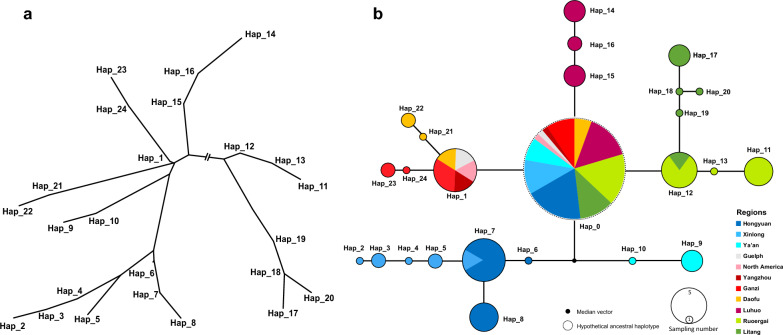
Phylogeny and network map of *Eimeria zuernii* haplotypes inferred on the basis of 54 *cox*1 data. **a** Topology tree of 24 haplotypes of *E. zuernii*. **b** Network map of *E. zuernii* haplotypes. *Eimeria zuernii* isolates from different geographic origins are shown in dark blue (Hongyuan), blue (Xinlong), light blue (Ya’an), gray (Guelph), pink (North America), dark red (Yangzhou), red (Ganzi), yellow (Daofu), purple (Luhuo), green (Ruoergai), and dark green (Litang). The small black dot represents the median vector, indicating unsampled or extinct haplotypes. The dotted cycle denotes the hypothetically ancestral haplotype. The proportion of haplotype frequencies is shown with sizable coils. The network branches linking the cycles show the relationships between the haplotypes

Based on 51 *E. zuernii* isolates (including the isolate for mitogenome sequencing in this study) and three reference isolates from GenBank, the mt *cox*1 sequences containing 31 variable sites (3.99%) defined 24 haplotypes of *E. zuernii*. The ML-based phylogenetic tree revealed that Ganzi population-specific haplotypes Hap_23 and Hap_24 closely clustered with Hap_1, and then three haplotypes together were paraphyletic with haplotypes of Daofu (Hap_21 and Hap_22), Ya’an (Hap_9 and Hap_10), LuHuo (Hap_14–16), and Xinlong plus Hongyuan (Hap_2–8) (Fig. 5a). It was also clear that Litang population-specific haplotypes Hap_17–20 were more closely related to Ruoergai population-specific haplotypes Hap_11–13 than others. These relationships among these haplotypes were further confirmed using the Network analysis. As shown in Fig. 5b, the network map revealed a radial-shaped clustering (RSC) with Hap_0 as the center, which was hypothetically inferred as the ancestral haplotype. It seemed that the haplotypes in each geographic population were highly specific. Although Hap_1 was shared by Ganzi, Daofu, and Yangzhou populations, Hap_12 was shared by Ruoergai and Litang populations, and Hap_7 was shared by Hongyuan and Xinlong populations. Moreover, *E. zuernii* from LuHuo and Ya’an appeared to form their own populations, and the latter was inferred as a possibly unsampled or extinct population because of the presence of a median vector (Fig. 5b). In general, network analysis is regarded as a better approach for representing genealogical relationships at a population level than traditional phylogenetic analysis because this approach is able to consider several factors related to intraspecific gene evolution, including the persistence of ancestral haplotypes, the existence of multiple descendant haplotypes, and low levels of sequence variation [58, 59]. Combined, the phylogenetic and network results suggested a low genetic structure among yak *E. zuernii* populations although they were prevalent in different geographic ranges of China.

## Conclusions

In this study, we sequenced the entire mitogenome of *E. zuernii* from the yak and characterized its genetic diversity across eight geographic ranges in China. Comparative mitogenomics determined both *cyt*b and *cox*3 genes as the most and least conserved PCGs, respectively, within the genus *Eimeria*. Evolutionary and phylogenetic analyses indicated that *E. zuernii* shared the closest relationship with *E. mephitidis*, and all species of *Eimeria* can be grouped into three clades according to their host origins. Further *cox*1-based genetic structure inference revealed 24 haplotypes of *E. zuernii* with high haplotype diversities and low nucleotide diversities, suggesting a low genetic structure and rapid evolutionary rate as well as a previous expansion event among *E. zuernii* populations. Future additional samplings could possibly uncover the geographic structure of *E. zuernii* in more detail.

### Supplementary Information

**Additional file 1. Table S1.** *Eimeria zuernii* isolates sequenced in this study.

**Additional file 2.** **Figure S1.** Multi-sequence comparisons of the ribosomal ITS-1 from the yak *Eimeria *and the congeneric *Eimeria zuernii*. 99.2%–100% identities are observed between* E. zuernii* and 51 *Eimeria* isolates identified in this study.

**Additional file 3.** **Table S2.** Summary of the mitogenome information of *Eimeria* spp. included in this study.

**Additional file 4.** **Table S3.** Organization of the *Eimeria zuernii* mitogenome.

**Additional file 5.** **Table S4.** Nucleotide composition of the complete *Eimeria zuernii* mitogenome.

**Additional file 6.** **Table S5.** Pairwise genetic divergences of *Eimeria* parasites based on mitogenome datasets.

**Additional file 7.** **Table S6.** Ka, Ks, and Ka/Ks ratios of three mt PCGs of *Eimeria zuernii *and congeneric species sequenced so far.

**Additional file 8.** **Table S7.** Wrights Fixation Index (Fst) values of eight geographic populations of *Eimeria zuernii *based on *cox*1 datasets.

## Data Availability

Molecular data have been deposited to GenBank with the following accession numbers: OQ476205 for *Eimeria zuernii* mitogenome, OR351547–OR351597 for *E. zuernii* ITS-1 dataset and OR039219–OR039268 for *E. zuernii cox*1 dataset. Fifty-one* E. zuernii* isolate specimens including the one for mitogenome sequencing in this study have been deposited at the Parasitological Museum of Sichuan Agricultural University (Sichuan, China) under the collection numbers XY2022_28–XY2022_78.

## Abbreviations

18S: Small-subunit ribosomal RNA
ITS-1: Internal transcribed spacer 1
rDNA: Nuclear ribosomal DNA
mtDNA: Mitogenomic DNA
mt: Mitochondrial
MP: Maximum parsimony
ML: Maximum likelihood
BI: Bayesian inference
PCG: Protein-coding gene
tRNA: Tranfer RNA
rRNA: Ribosomal RNA
LSU: Large subunit ribosomal RNA
SSU: Small subunit ribosomal RNA
*cox*1: Cytochrome c oxidase subunit I
*cox*3: Cytochrome c oxidase subunit III
*cyt*b: Cytochrome b
Fst: Wright’s fixation index
Ka: Non-synonymous substitutions
Ks: Synonymous substitutions
RSCU: Relative synonymous codon usage

## References

[1] Bangoura B, Bardsley KD (2020). Ruminant coccidiosis. Vet Clin North Am Food Anim Pract.

[2] Li DL, Gong QL, Ge GY, Wang Q, Sheng CY, Ma BY (2021). Prevalence and infection risk factors of bovine *Eimeria* in China: a systematic review and meta-analysis. Parasite.

[3] Bangoura B, Daugschies A (2007). Parasitological and clinical parameters of experimental *Eimeria zuernii* infection in calves and influence on weight gain and haemogram. Parasitol Res.

[4] von Samson-Himmelstjerna G, Epe C, Wirtherle N, von der Heyden V, Welz C, Radeloff I (2006). Clinical and epidemiological characteristics of *Eimeria* infections in first-year grazing cattle. Vet Parasitol.

[5] Ekawasti F, Nurcahyo W, Wardhana AH, Shibahara T, Matsubayashi M (2019). Molecular characterization of highly pathogenic *Eimeria* species among beef cattle on Java Island, Indonesia. Parasitol Int.

[6] Wang X, Pei J, Bao P, Cao M, Guo S, Song R (2021). Mitogenomic diversity and phylogeny analysis of yak (*Bos grunniens*). BMC Genomics.

[7] Bangoura B, Mundt HC, Schmäschke R, Westphal B, Daugschies A (2012). Prevalence of *Eimeria bovis* and *Eimeria zuernii* in German cattle herds and factors influencing oocyst excretion. Parasitol Res.

[8] David OFS, Rabiu M, Karimat H, Sanda IM, Ganiyu IA (2020). Epidemiological studies of *Eimeria* species of cattle in Ilorin, North-Central Nigeria. Ann Parasitol.

[9] Griffith SM, Gigley J, Fox J, Bangoura B. Identification and characterization of *Eimeria* spp. in western north American Bison (*Bison bison*) herds and potential risk of cross-species transmission. Vet Parasitol Reg Stud Rep. 2021;26:100627. 10.1016/j.vprsr.2021.100627 34879938

[10] Lopez-Osorio S, Villar D, Failing K, Taubert A, Hermosilla C, Chaparro-Gutierrez JJ (2020). Epidemiological survey and risk factor analysis on *Eimeria* infections in calves and young cattle up to 1 year old in Colombia. Parasitol Res.

[11] Kawahara F, Zhang G, Mingala CN, Tamura Y, Nunoya T (2010). Genetic analysis and development of species-specific PCR assays based on ITS-1 region of rRNA in bovine *Eimeria* parasites. Vet Parasitol.

[12] Koreeda T, Kawakami T, Okada A, Hirashima Y, Imai N, Sasai K (2017). Pathogenic characteristics of a novel intranuclear coccidia in Japanese black calves and its genetic identification as *Eimeria subspherica*. Parasitol Res.

[13] Pyziel AMD Aleksander W Klich, DanielLaskowski, Zdzislaw. A morphological and molecular comparison of *Eimeria bovis*-like oocysts (Apicomplexa: Eimeriidae) from European bison, *Bison bonasus* L., and cattle, *Bos taurus* L., and the development of two multiplex PCR assays for their identification. Vet Parasitol. 2019;275:108917. 10.1016/j.vetpar.2019.08.011 31473050

[14] Brown WM, George M, Wilson AC (1979). Rapid evolution of animal mitochondrial DNA. Proc Natl Acad Sci U S A.

[15] Awadi A (2017). Host species and pathogenicity effects in the evolution of the mitochondrial genomes of *Eimeria* species (Apicomplexa; Coccidia; Eimeriidae). J Biol Res (Thessalon).

[16] Hastutiek P, Lastuti NDR, Suwanti LT, Sunarso A, Suprihati E, Kurniawati DA (2022). Coproparasitological examinations and molecular determination of *Eimeria* species in Madura cattle reared on Madura Island. Indonesia Parasitol Int.

[17] Morgan JAT, Godwin RM (2017). Mitochondrial genomes of Australian chicken *Eimeria* support the presence of ten species with low genetic diversity among strains. Vet Parasitol.

[18] Ogedengbe JD, Hanner RH, Barta JR (2011). DNA barcoding identifies *Eimeria* species and contributes to the phylogenetics of coccidian parasites (Eimeriorina, Apicomplexa, Alveolata). Int J Parasitol.

[19] Ogedengbe ME, El-Sherry S, Ogedengbe JD, Chapman HD, Barta JR (2018). Phylogenies based on combined mitochondrial and nuclear sequences conflict with morphologically defined genera in the eimeriid coccidia (Apicomplexa). Int J Parasitol.

[20] Snyder RP, Guerin MT, Hargis BM, Imai R, Kruth PS, Page G (2021). Exploiting digital droplet PCR and Next Generation Sequencing technologies to determine the relative abundance of individual *Eimeria* species in a DNA sample. Vet Parasitol.

[21] Amambua-Ngwa A, Tetteh KKA, Manske M, Gomez-Escobar N, Stewart LB, Deerhake ME (2012). Population genomic scan for candidate signatures of balancing selection to guide antigen characterization in malaria parasites. PLoS Genet.

[22] Minot S, Melo MB, Li F, Lu D, Niedelman W, Levine SS (2012). Admixture and recombination among *Toxoplasma gondii* lineages explain global genome diversity. Proc Natl Acad Sci U S A.

[23] Blake DP, Worthing K, Jenkins MC (2020). Exploring *Eimeria* genomes to understand population biology: recent progress and future opportunities. Genes.

[24] Gorton MJ, Kasl EL, Detwiler JT, Criscione CD (2012). Testing local-scale panmixia provides insights into the cryptic ecology, evolution, and epidemiology of metazoan animal parasites. Parasitol.

[25] Huyse T, Poulin R, Théron A (2005). Speciation in parasites: a population genetics approach. Trends Parasitol.

[26] Myers N, Mittermeier RA, Mittermeier CG, da Fonseca GA, Kent J (2000). Biodiversity hotspots for conservation priorities. Nature.

[27] Florião MM, Lopes B do B, Berto BP, Lopes CWG. New approaches for morphological diagnosis of bovine *Eimeria* species: a study on a subtropical organic dairy farm in Brazil. Trop Anim Health Prod. 2016;48:577–84. 10.1007/s11250-016-0998-5 26873157

[28] Hahn C, Bachmann L, Chevreux B (2013). Reconstructing mitochondrial genomes directly from genomic next-generation sequencing reads—a baiting and iterative mapping approach. Nucleic Acids Res.

[29] Perna NT, Kocher TD (1995). Patterns of nucleotide composition at fourfold degenerate sites of animal mitochondrial genomes. J Mol Evol.

[30] Srivathsan A, Meier R (2012). On the inappropriate use of Kimura-2-parameter (K2P) divergences in the DNA-barcoding literature. Cladistics.

[31] Zhang Z, Li J, Zhao XQ, Wang J, Wong GKS, Yu J (2006). KaKs_Calculator: calculating Ka and Ks through model selection and model averaging. Genom Proteom Bioinf.

[32] Katoh K, Misawa K, Kuma K, Miyata T (2002). MAFFT: a novel method for rapid multiple sequence alignment based on fast Fourier transform. Nucleic Acids Res.

[33] Goloboff PA, Catalano SA, Torres A (2022). Parsimony analysis of phylogenomic datasets (II): evaluation of PAUP*. MEGA and MPBoot Cladistics.

[34] Guindon S, Gascuel O (2003). A simple, fast, and accurate algorithm to estimate large phylogenies by maximum likelihood. Syst Biol.

[35] Ronquist F, Teslenko M, van der Mark P, Ayres DL, Darling A, Höhna S, et al. MrBayes 3.2: efficient Bayesian phylogenetic inference and model choice across a large model space. Syst Biol. 2012;61:539–42. 10.1093/sysbio/sys029 PMC3329765 22357727

[36] Kalyaanamoorthy S, Minh BQ, Wong TKF, von Haeseler A, Jermiin LS (2017). ModelFinder: fast model selection for accurate phylogenetic estimates. Nat Methods.

[37] Hafeez MA, Vrba V, Barta JR (2016). The complete mitochondrial genome sequence of *Eimeria innocua* (Eimeriidae, Coccidia, Apicomplexa). Mitochondrial DNA A DNA Mapp Seq Anal.

[38] Liu G, Li Q, Wang C, Xu C (2019). The complete mitochondrial genome of *Eimeria anseris* from the wintering greater white-fronted goose in Shengjin Lake, China, and phylogenetic relationships among *Eimeria* species. Parasitol Res.

[39] Ogedengbe ME, El-Sherry S, Whale J, Barta JR (2014). Complete mitochondrial genome sequences from five *Eimeria* species (Apicomplexa; Coccidia; Eimeriidae) infecting domestic turkeys. Parasit Vectors.

[40] Feagin JE, Harrell MI, Lee JC, Coe KJ, Sands BH, Cannone JJ (2012). The fragmented mitochondrial ribosomal RNAs of *Plasmodium falciparum*. PLoS ONE.

[41] Rejman EE, Kehoe R, Barta JR (2021). The complete mitochondrial genome sequence of *Eimeria leuckarti* (Eimeriidae, Coccidia, Apicomplexa) infecting domestic horses (*Equus ferus caballus*). Mitochondrial DNA B Resour.

[42] Tian SQ, Cui P, Fang SF, Liu GH, Wang CR, Zhu XQ (2015). The complete mitochondrial genome sequence of *Eimeria magna* (Apicomplexa: Coccidia). Mitochondrial DNA.

[43] Zhou X, Wang L, Zhu P, Yang Z, Wang Z, Chen Y (2023). Comprehensive molecular characterization of complete mitogenome assemblies of 33 *Eimeria* isolates infecting domestic chickens. Parasit Vectors.

[44] Hikosaka K, Watanabe Y, Tsuji N, Kita K, Kishine H, Arisue N (2010). Divergence of the mitochondrial genome structure in the apicomplexan parasites, *Babesia* and *Theileria*. Mol Biol Evol.

[45] Perkins SL (2008). Molecular systematics of the three mitochondrial protein-coding genes of malaria parasites: corroborative and new evidence for the origins of human malaria. Mitochondrial DNA.

[46] Petersen HH, Yang R, Chriél M, Hansen MS, Ryan UM (2018). Morphological and molecular characterisation of *Eimeria vison*-like oocysts (Apicomplexa: Eimeriidae) in farmed mink (*Neovison vison*) in Denmark. Parasitol Res.

[47] Tang K, Guo Y, Zhang L, Rowe LA, Roellig DM, Frace MA, et al. Genetic similarities between *Cyclospora cayetanensis* and cecum-infecting avian *Eimeria* spp. in apicoplast and mitochondrial genomes. Parasit Vectors. 2015;8:358. 10.1186/s13071-015-0966-3 PMC4495940 26152563

[48] Vrba V, Pakandl M (2015). Host specificity of turkey and chicken *Eimeria*: controlled cross-transmission studies and a phylogenetic view. Vet Parasitol.

[49] Zhang K, Liang G, Lang J, Qin Z, Zhang Y, Wang Y, et al. *Eimeria* spp. (Eimeriidae) in the migratory whooper swan (*Cygnus cygnus*) Linnaeus, 1758 (Anatidae) from Sanmenxia Swan Lake National Urban Wetland Park in the middle reaches of the Yellow River in China. Parasitol Res. 2022;121:2967–77. 10.1007/s00436-022-07629-x 35986168

[50] Silva LMR, Chávez-Maya F, Macdonald S, Pegg E, Blake DP, Taubert A (2017). A newly described strain of *Eimeria arloingi* (strain A) belongs to the phylogenetic group of ruminant-infecting pathogenic species, which replicate in host endothelial cells *in vivo*. Vet Parasitol.

[51] Vermeulen ET, Lott MJ, Eldridge MDB, Power ML (2016). Evaluation of next generation sequencing for the analysis of *Eimeria* communities in wildlife. J Microbiol Methods.

[52] Thomas WK, Wilson AC (1991). Mode and tempo of molecular evolution in the nematode *Caenorhabditis*: cytochrome oxidase II and calmodulin sequences. Genetics.

[53] Mao M, Liu HL (2015). Genetic diversity of *Trichomonas vaginalis* clinical isolates from Henan province in central China. Pathog Glob Health.

[54] Nehra AK, Kumari A, Kundave VR, Vohra S, Ram H (2022). Molecular insights into the population structure and haplotype network of *Theileria annulata* based on the small-subunit ribosomal RNA (18S rRNA) gene. Infect Genet Evol.

[55] Zhang X, Dan J, Wang L, Liu H, Zhou Z, Ma X (2021). High genetic diversity of *Giardia duodenalis* assemblage E in Chinese dairy cattle. Infect Genet Evol.

[56] Zhang C, Yang R, Wu L, Luo C, Guo X, Deng Y (2021). Molecular phylogeny of the *Anopheles hyrcanus* group (Diptera: Culicidae) based on rDNA-ITS2 and mtDNA-COII. Parasit Vectors.

[57] Zhong Z, Dan J, Yan G, Tu R, Tian Y, Cao S (2018). Occurrence and genotyping of *Giardia duodenalis* and *Cryptosporidium* in pre-weaned dairy calves in central Sichuan province. China Parasite.

[58] Zhou X, Xie Y, Zhang Z, Wang C, Sun Y, Gu X (2013). Analysis of the genetic diversity of the nematode parasite *Baylisascaris schroederi* from wild giant pandas in different mountain ranges in China. Parasit Vectors.

[59] Clark EL, Tomley FM, Blake DP (2017). Are *Eimeria* genetically diverse, and does it matter?. Trends Parasitol.

