# Supplementary material for: Eimeria zuernii (Eimeriidae: Coccidia): mitochondrial genome and genetic diversity in the Chinese yak
Source: Parasit Vectors. 2023 Sep 3;16:312. doi: 10.1186/s13071-023-05925-8 (PMC10475197; doi:10.1186/s13071-023-05925-8)
Supplement: Supplementary file 2 — Additional file 2. Figure S1. Multi-sequence comparisons of the ribosomal ITS-1 from the yak Eimeria and the congeneric Eimeria zuernii. 99.2%–100% identities are observed between E. zuernii and 51 Eimeria isolates identified in this study. [file 13071_2023_5925_MOESM2_ESM.pdf]

99.2%-100% identities

```
Eimeria zuernii/AB769665  CTGTTGTGGATAGTTACTGTTGTGTTTGTTCCCTTATGGCATTTCCAAGACTGTGGTCTTATAAAAAACCTCACCACAAAACCCTGTAAACTATTGATTCTTTCAGTTCCGGTTTTTTTTT   121
GZ_mtDNA/OR351547         CTGTTGTGGATAGTTACTGTTGTGTTTGTTCCCTTATGGCATTTCCAAGACTGTGGTCTTATAAAAAACCTCACCACAAAACCCTGTAAACTATTGATTCTTTCAGTTCCGGTTTTTTTTT   121
XL_1/OR351548             CTGTTGTGGATAGTTACTGTTGTGTTTGTTCCCTTATGGCATTTCCAAGACTGTGGTCTTATAAAAAACCTCACCACAAAACCCTGTAAACTATTGATTCTTTCAGTTCCGGTTTTTTTTT   121
XL_2/OR351549             CTGTTGTGGATAGTTACTGTTGTGTTTGTTCCCTTATGGCATTTCCAAGACTGTGGTCTTATAAAAAACCTCACCACAAAACCCTGTAAACTATTGATTCTTTCAGTTCCGGTTTTTTTTT   121
XL_3/OR351550             CTGTTGTGGATAGTTACTGTTGTGTTTGTTCCCTTATGGCATTTCCAAGACTGTGGTCTTATAAAAAACCTCACCACAAAACCCTGTAAACTATTGATTCTTTCAGTTCCGGTTTTTTTTT   121
XL_4/OR351551             CTGTTGTGGATAGTTACTGTTGTGTTTGTTCCCTTATGGCATTTCCAAGACTGTGGTCTTATAAAAAACCTCACCACAAAACCCTGTAAACTATTGATTCTTTCAGTTCCGGTTTTTTTTT   121
XL_5/OR351552             CTGTTGTGGATAGTTACTGTTGTGTTTGTTCCCTTATGGCATTTCCAAGACTGTGGTCTTATAAAAAACCTCACCACAAAACCCTGTAAACTATTGATTCTTTCAGTTCCGGTTTTTTTTT   121
XL_6/OR351553             CTGTTGTGGATAGTTACTGTTGTGTTTGTTCCCTTATGGCATTTCCAAGACTGTGGTCTTATAAAAAACCTCACCACAAAACCCTGTAAACTATTGATTCTTTCAGTTCCGGTTTTTTTTT   121
HY_1/OR351554             CTGTTGTGGATAGTTACTGTTGTGTTTGTTCCCTTATGGCATTTCCAAGACTGTGGTCTTATAAAAAACCTCACCACAAAACCCTCTAAACTATTGATTCTTTCAGTTCCGGTTTTTTTTT   121
HY_2/OR351555             CTGTTGTGGATAGTTACTGTTGTGTTTGTTCCCTTATGGCATTTCCAAGACTGTGGTCTTATAAAAAACCTCACCACAAAACCCTCTAAACTATTGATTCTTTCAGTTCCGGTTTTTTTTT   121
HY_3/OR351556             CTGTTGTGGATAGTTACTGTTGTGTTTGTTCCCTTATGGCATTTCCAAGACTGTGGTCTTATAAAAAACCTCACCACAAAACCCTCTAAACTATTGATTCTTTCAGTTCCGGTTTTTTTTT   121
HY_4/OR351557             CTGTTGTGGATAGTTACTGTTGTGTTTGTTCCCTTATGGCATTTCCAAGACTGTGGTCTTATAAAAAACCTCACCACAAAACCCTCTAAACTATTGATTCTTTCAGTTCCGGTTTTTTTTT   121
HY_5/OR351558             CTGTTGTGGATAGTTACTGTTGTGTTTGTTCCCTTATGGCATTTCCAAGACTGTGGTCTTATAAAAAACCTCACCACAAAACCCTCTAAACTATTGATTCTTTCAGTTCCGGTTTTTTTTT   121
HY_6/OR351559             CTGTTGTGGATAGTTACTGTTGTGTTTGTTCCCTTATGGCATTTCCAAGACTGTGGTCTTATAAAAAACCTCACCACAAAACCCTGTAAACTATTGATTCTTTCAGTTCCGGTTTTTTTTT   121
HY_7/OR351560             CTGTTGTGGATAGTTACTGTTGTGTTTGTTCCCTTATGGCATTTCCAAGACTGTGGTCTTATAAAAAACCTCACCACAAAACCCTGTAAACTATTGATTCTTTCAGTTCCGGTTTTTTTTT   121
HY_8/OR351561             CTGTTGTGGATAGTTACTGTTGTGTTTGTTCCCTTATGGCATTTCCAAGACTGTGGTCTTATAAAAAACCTCACCACAAAACCCTGTAAACTATTGATTCTTTCAGTTCCGGTTTTTTTTT   121
HY_9/OR351562             CTGTTGTGGATAGTTACTGTTGTGTTTGTTCCCTTATGGCATTTCCAAGACTGTGGTCTTATAAAAAACCTCACCACAAAACCCTGTAAACTATTGATTCTTTCAGTTCCGGTTTTTTTTT   121
HY_10/OR351563            CTGTTGTGGATAGTTACTGTTGTGTTTGTTCCCTTATGGCATTTCCAAGACTGTGGTCTTATAAAAAACCTCACCACAAAACCCTGTAAACTATTGATTCTTTCAGTTCCGGTTTTTTTTT   121
YA_1/OR351564             CTGTTGTGGATAGTTACTGTTGTGTTTGTTCCCTTATGGCATTTCCAAGACTGTGGTCTTATAAAAAACCTCACCACAAAACCCTGTAAACTATTGATTCTTTCAGTTCCGGTTTTTTTTT   121
YA_2/OR351565             CTGTTGTGGATAGTTACTGTTGTGTTTGTTCCCTTATGGCATTTCCAAGACTGTGGTCTTATAAAAAACCTCACCACAAAACCCTGTAAACTATTGATTCTTTCAGTTCCGGTTTTTTTTT   121
YA_3/OR351566             CTGTTGTGGATAGTTACTGTTGTGTTTGTTCCCTTATGGCATTTCCAAGACTGTGGTCTTATAAAAAACCTCACCACAAAACCCTGTAAACTATTGATTCTTTCAGTTCCGGTTTTTTTTT   121
YA_4/OR351567             CTGTTGTGGATAGTTACTGTTGTGTTTGTTCCCTTATGGCATTTCCAAGACTGTGGTCTTATAAAAAACCTCACCACAAAACCCTGTAAACTATTGATTCTTTCAGTTCCGGTTTTTTTTT   121
REG_1/OR351568            CTGTTGTGGATAGTTACTGTTGTGTTTGTTCCCTTATGGCATTTCCAAGACTGTGGTCTTATAAAAAACCTCACCACAAAACCCTCTAAACTATTGATTCTTTCAGTTCCGGTTTTTTTTT   121
REG_2/OR351569            CTGTTGTGGATAGTTACTGTTGTGTTTGTTCCCTTATGGCATTTCCAAGACTGTGGTCTTATAAAAAACCTCACCACAAAACCCTCTAAACTATTGATTCTTTCAGTTCCGGTTTTTTTTT   121
REG_3/OR351570            CTGTTGTGGATAGTTACTGTTGTGTTTGTTCCCTTATGGCATTTCCAAGACTGTGGTCTTATAAAAAACCTCACCACAAAACCCTGTAAACTATTGATTCTTTCAGTTCCGGTTTTTTTTT   121
REG_4/OR351571            CTGTTGTGGATAGTTACTGTTGTGTTTGTTCCCTTATGGCATTTCCAAGACTGTGGTCTTATAAAAAACCTCACCACAAAACCCTCTAAACTATTGATTCTTTCAGTTCCGGTTTTTTTTT   121
REG_5/OR351572            CTGTTGTGGATAGTTACTGTTGTGTTTGTTCCCTTATGGCATTTCCAAGACTGTGGTCTTATAAAAAACCTCACCACAAAACCCTGTAAACTATTGATTCTTTCAGTTCCGGTTTTTTTTT   121
REG_6/OR351573            CTGTTGTGGATAGTTACTGTTGTGTTTGTTCCCTTATGGCATTTCCAAGACTGTGGTCTTATAAAAAACCTCACCACAAAACCCTGTAAACTATTGATTCTTTCAGTTCCGGTTTTTTTTT   121
REG_7/OR351574            CTGTTGTGGATAGTTACTGTTGTGTTTGTTCCCTTATGGCATTTCCAAGACTGTGGTCTTATAAAAAACCTCACCACAAAACCCTCTAAACTATTGATTCTTTCAGTTCCGGTTTTTTTTT   121
REG_8/OR351575            CTGTTGTGGATAGTTACTGTTGTGTTTGTTCCCTTATGGCATTTCCAAGACTGTGGTCTTATAAAAAACCTCACCACAAAACCCTGTAAACTATTGATTCTTTCAGTTCCGGTTTTTTTTT   121
REG_9/OR351576            CTGTTGTGGATAGTTACTGTTGTGTTTGTTCCCTTATGGCATTTCCAAGACTGTGGTCTTATAAAAAACCTCACCACAAAACCCTGTAAACTATTGATTCTTTCAGTTCCGGTTTTTTTTT   121
LH_1/OR351577             CTGTTGTGGATAGTTACTGTTGTGTTTGTTCCCTTATGGCATTTCCAAGACTGTGGTCTTATAAAAAACCTCACCACAAAACCCTGTAAACTATTGATTCTTTCAGTTCCGGTTTTTTTTT   121
LH_2/OR351578             CTGTTGTGGATAGTTACTGTTGTGTTTGTTCCCTTATGGCATTTCCAAGACTGTGGTCTTATAAAAAACCTCACCACAAAACCCTGTAAACTATTGATTCTTTCAGTTCCGGTTTTTTTTT   121
LH_3/OR351579             CTGTTGTGGATAGTTACTGTTGTGTTTGTTCCCTTATGGCATTTCCAAGACTGTGGTCTTATAAAAAACCTCACCACAAAACCCTGTAAACTATTGATTCTTTCAGTTCCGGTTTTTTTTT   121
LH_4/OR351580             CTGTTGTGGATAGTTACTGTTGTGTTTGTTCCCTTATGGCATTTCCAAGACTGTGGTCTTATAAAAAACCTCACCACAAAACCCTGTAAACTATTGATTCTTTCAGTTCCGGTTTTTTTTT   121
LH_5/OR351581             CTGTTGTGGATAGTTACTGTTGTGTTTGTTCCCTTATGGCATTTCCAAGACTGTGGTCTTATAAAAAACCTCACCACAAAACCCTGTAAACTATTGATTCTTTCAGTTCCGGTTTTTTTTT   121
LH_6/OR351582             CTGTTGTGGATAGTTACTGTTGTGTTTGTTCCCTTATGGCATTTCCAAGACTGTGGTCTTATAAAAAACCTCACCACAAAACCCTGTAAACTATTGATTCTTTCAGTTCCGGTTTTTTTTT   121
LH_7/OR351583             CTGTTGTGGATAGTTACTGTTGTGTTTGTTCCCTTATGGCATTTCCAAGACTGTGGTCTTATAAAAAACCTCACCACAAAACCCTGTAAACTATTGATTCTTTCAGTTCCGGTTTTTTTTT   121
LH_8/OR351584             CTGTTGTGGATAGTTACTGTTGTGTTTGTTCCCTTATGGCATTTCCAAGACTGTGGTCTTATAAAAAACCTCACCACAAAACCCTGTAAACTATTGATTCTTTCAGTTCCGGTTTTTTTTT   121
LT_1/OR351585             CTGTTGTGGATAGTTACTGTTGTGTTTGTTCCCTTATGGCATTTCCAAGACTGTGGTCTTATAAAAAACCTCACCACAAAACCCTGTAAACTATTGATTCTTTCAGTTCCGGTTTTTTTTT   121
LT_2/OR351586             CTGTTGTGGATAGTTACTGTTGTGTTTGTTCCCTTATGGCATTTCCAAGACTGTGGTCTTATAAAAAACCTCACCACAAAACCCTGTAAACTATTGATTCTTTCAGTTCCGGTTTTTTTTT   121
LT_3/OR351587             CTGTTGTGGATAGTTACTGTTGTGTTTGTTCCCTTATGGCATTTCCAAGACTGTGGTCTTATAAAAAACCTCACCACAAAACCCTGTAAACTATTGATTCTTTCAGTTCCGGTTTTTTTTT   121
LT_4/OR351588             CTGTTGTGGATAGTTACTGTTGTGTTTGTTCCCTTATGGCATTTCCAAGACTGTGGTCTTATAAAAAACCTCACCACAAAACCCTGTAAACTATTGATTCTTTCAGTTCCGGTTTTTTTTT   121
LT_5/OR351589             CTGTTGTGGATAGTTACTGTTGTGTTTGTTCCCTTATGGCATTTCCAAGACTGTGGTCTTATAAAAAACCTCACCACAAAACCCTGTAAACTATTGATTCTTTCAGTTCCGGTTTTTTTTT   121
LT_6/OR351590             CTGTTGTGGATAGTTACTGTTGTGTTTGTTCCCTTATGGCATTTCCAAGACTGTGGTCTTATAAAAAACCTCACCACAAAACCCTGTAAACTATTGATTCTTTCAGTTCCGGTTTTTTTTT   121
DF_1/OR351591             CTGTTGTGGATAGTTACTGTTGTGTTTGTTCCCTTATGGCATTTCCAAGACTGTGGTCTTATAAAAAACCTCACCACAAAACCCTGTAAACTATTGATTCTTTCAGTTCCGGTTTTTTTTT   121
DF_2/OR351592             CTGTTGTGGATAGTTACTGTTGTGTTTGTTCCCTTATGGCATTTCCAAGACTGTGGTCTTATAAAAAACCTCACCACAAAACCCTGTAAACTATTGATTCTTTCAGTTCCGGTTTTTTTTT   121
DF_3/OR351593             CTGTTGTGGATAGTTACTGTTGTGTTTGTTCCCTTATGGCATTTCCAAGACTGTGGTCTTATAAAAAACCTCACCACAAAACCCTGTAAACTATTGATTCTTTCAGTTCCGGTTTTTTTTT   121
GZ_1/OR351594             CTGTTGTGGATAGTTACTGTTGTGTTTGTTCCCTTATGGCATTTCCAAGACTGTGGTCTTATAAAAAACCTCACCACAAAACCCTGTAAACTATTGATTCTTTCAGTTCCGGTTTTTTTTT   121
GZ_2/OR351595             CTGTTGTGGATAGTTACTGTTGTGTTTGTTCCCTTATGGCATTTCCAAGACTGTGGTCTTATAAAAAACCTCACCACAAAACCCTGTAAACTATTGATTCTTTCAGTTCCGGTTTTTTTTT   121
GZ_3/OR351596             CTGTTGTGGATAGTTACTGTTGTGTTTGTTCCCTTATGGCATTTCCAAGACTGTGGTCTTATAAAAAACCTCACCACAAAACCCTGTAAACTATTGATTCTTTCAGTTCCGGTTTTTTTTT   121
GZ_4/OR351597             CTGTTGTGGATAGTTACTGTTGTGTTTGTTCCCTTATGGCATTTCCAAGACTGTGGTCTTATAAAAAACCTCACCACAAAACCCTGTAAACTATTGATTCTTTCAGTTCCGGTTTTTTTTT   121
Consensus                 ctgttgtggatagttactgttgtgtttgttcccttatggcatttccaagactgtggtcttataaaaaacctcaccacaaaaccct taaactattgattctttcagttccggttttttttt
```

Figure S1. Multi-sequence comparisons of the ribosomal ITS-1 from the yak *Eimeria* and the congeneric *Eimeria zuernii*. 99.2%-100% identities are observed between *E. zuernii* and 51 *Eimeria* isolates identified in this study.
